# miR-let-7b and miR-let-7c suppress tumourigenesis of human mucosal melanoma and enhance the sensitivity to chemotherapy

**DOI:** 10.1186/s13046-019-1190-3

**Published:** 2019-05-22

**Authors:** Huan Tang, Meng Ma, Jie Dai, Chuanliang Cui, Lu Si, Xinan Sheng, Zhihong Chi, Longwen Xu, Sifan Yu, Tianxiao Xu, Junya Yan, Huan Yu, Lu Yang, Yan Kong, Jun Guo

**Affiliations:** Department of Renal Cancer and Melanoma, Peking University Cancer Hospital & Institute, 52 Fucheng Road, Beijing, 100142 China

**Keywords:** miR-let-7b, miR-let-7c, Mucosal melanoma, Chemotherapy

## Abstract

**Background:**

Mucosal melanoma with poor prognosis is a common histopathologic subtype of melanoma among Chinese and other Asian peoples. Regulated microRNAs (miRNAs) have been reported as oncogenes or tumour suppressors in melanoma. However, the roles of specific miRNAs in mucosal melanoma remain largely unknown. Here, we aimed to assess the biological functions, molecular mechanisms and clinical potential of miR-let-7b and miR-let-7c in mucosal melanoma.

**Methods:**

The expression of miR-let-7b and miR-let-7c in mucosal melanoma was determined by quantitative polymerase chain reaction (qPCR). Cutoff scores for miR-let-7b and miR-let-7c expressions were calculated through receiver operating characteristic (ROC) curve analysis in 106 mucosal melanoma patients according to recurrence. Correlations of miR-let-7b and miR-let-7c expression with clinicopathological characteristics, disease-free survival (DFS) and clinical benefits after treatment were then statistically analysed. The biological functions and molecular mechanisms of miR-let-7b and miR-let-7c were studied in vitro and in vivo.

**Results:**

The expression of miR-let-7b and miR-let-7c was decreased in 94 cases (88.7%) and 89 cases (84.0%) of 106 mucosal melanoma patients compared with mucosal nevi. A correlation was observed between the expression of miR-let-7b, miR-let-7c and DFS after surgery. In addition, overexpression of miR-let-7b or miR-let-7c inhibited mucosal melanoma cell growth, migration, invasion and metastasis and induced cell apoptosis and cell cycle arrest in vitro and in vivo. Mechanistically, miR-let-7b and miR-let-7c directly targeted metadherin (MTDH) and calumenin (CALU) and suppressed phospho-ERK in mucosal melanoma cells. MTDH and CALU reversed the partial function of miR-let-7b and miR-let-7c in vitro. Furthermore, progression-free survival (PFS) of mucosal melanoma patients upon temozolomide-based and paclitaxel-based chemotherapy was related to miR-let-7b and miR-let-7c expression. Overexpression of miR-let-7b or miR-let-7c in patient-derived xenograft (PDX) models and certain mucosal melanoma cells had better growth inhibition after temozolomide and paclitaxel treatment. MTDH reversed the sensitivity of miR-let-7b and miR-let-7c to paclitaxel in vitro.

**Conclusions:**

Our results suggested that miR-let-7b and miR-let-7c inhibited the recurrence of mucosal melanoma through inhibiting cell growth, migration, invasion and metastasis, inducing cell apoptosis and cell cycle arrest by targeting MTDH and CALU. In addition, miR-let-7b and miR-let-7c increased sensitivity to chemotherapeutic agents by targeting MTDH.

**Electronic supplementary material:**

The online version of this article (10.1186/s13046-019-1190-3) contains supplementary material, which is available to authorized users.

## Background

Melanoma is one of the most aggressive skin cancers, with poor prognosis. In 2018, 91,270 new melanoma cases and 9320 melanoma deaths are projected to occur in the United States [1]. Recent cancer statistics from China showed that the estimated numbers of new cases and deaths of melanoma were 8000 and 3200, respectively [2]. The incidence of melanoma is lower in China, but the death rate is dramatically higher. Unlike other tumours, melanoma has a wide range of pathologic subtypes that are related with ethnicity. The incidence of mucosal melanoma is significantly lower in Caucasians (1.3% of all melanomas) [3, 4], whereas this is the second most common subtype in Asia (22.6% of all melanomas) [5]. However, mucosal melanoma is more aggressive and it relapses and metastasizes more easily than other subtypes. The 5-year survival rates for patients with mucosal melanoma versus acral melanoma (the most common cutaneous melanoma in Asia) were 26.8% versus 53.9% [5]. The median survival time was 3.58 versus 4.67 years for patients with mucosal melanoma versus non-mucosal melanoma patients [5]. Thus, it is essential to further study mucosal melanoma to improve the survival of Chinese melanoma patients.

miRNAs are small noncoding RNAs of only 21–23 bp in length [6] that have a wide range of functions. Abnormal expression of miRNAs could lead to the occurrence of some diseases, including cancers [7–12]. Therefore, miRNAs have unique advantages and thus are selected for further study in mucosal melanoma. miR-let-7, originally found in *C. elegans* as heterochronic switch gene was the earliest identified miRNA [13] and is a widely studied miRNA in melanoma [14]. However, the role of miR-let-7 in mucosal melanoma remains largely unknown. Therefore, in this study, we aimed to assess the role of miR-let-7b and miR-let-7c in mucosal melanoma and further explore their biological functions and potential molecular mechanisms.

## Methods

### Mucosal melanoma tissues and cell lines

In total, 106 tissues of mucosal melanoma patients were obtained from the Renal Cancer and Melanoma Department of Beijing Cancer Hospital from 2012 to 2017. Clinicopathological characteristics of patients were collected from medical records. Two mucosal melanoma cell lines were used in the research. HMVII was obtained from Sigma-Aldrich Corporation and cultured in Ham’s F10 Nutrient Mix (Gibco BRL, Carlsbad, CA, USA) supplemented with 2 mM Glutamine (Gibco BRL, Carlsbad, CA, USA) and 15% fetal bovine serum (Gibco BRL, Carlsbad, CA, USA). GAK was purchased from the Japanese Collection of Research Bioresources (JCRB) Cell Bank and cultured in Ham’s F12 Nutrient Mix (Gibco BRL, Carlsbad, CA, USA) supplemented with 15% fetal bovine serum (Gibco BRL, Carlsbad, CA, USA). And all cells were incubated in a humidified 37 °C incubator supplemented with 5% CO2.

### RNA extraction, reverse transcription and expression analyses

Total RNA was extracted from tissues and cells using a mirVana™ miRNA Isolation Kit (Ambion, Carlsbad, CA, USA) according to the manufacturer’s protocols. RNA samples OD260/OD280 ratios ranging from 1.9–2.0 were considered good quality. ABI® Reverse Transcription Kit (Applied Biosystems, Foster City, CA) was used for reverse transcription. Quantitative polymerase chain reaction (PCR) measurements for miR-let-7b-5p and miR-let-7c-5p were performed using TaqMan MicroRNA Assays (Applied Biosystems, Foster City, CA). Relative expressions of miR-let-7b-5p and miR-let-7c-5p were calculated using a comparative CT method with an internal control RNU6B for miRNA. The mRNA expression of target genes was detected by microarray hybridization and gene expression analyses that were performed using the GeneChip Human Genome U133 Plus 2.0 microarrays (Affymetrix) and Affymetrix GeneChip System (Biotechnology, Shanghai, China).

### Transient transfection and virus infection

Cells were cultured in a 6-well plate (40000 cells/well) and transfected with transient miR-let-7b-5p, miR-let-7c-5p or negative control miRNA (RiboBio, Guangzhou, China) using Lipofectamine RNAiMAX (Invitrogen, Carlsbad, CA, USA). Transient plasmids of MTDH, CALU or negative control (Vigene Biosciences, Shandong, China) were transfected into cells with Lipofectamine 3000 (Invitrogen, Carlsbad, CA, USA). Stable miR-let-7b-5p or miR-let-7c-5p expressing cell lines and negative control cell lines were created with lentiviral infection (Hanbio, Shanghai, China) in a 6-well plate (20,000 cells/well). Transient transfection and virus infection were used in vitro and in vivo experiments, respectively.

### Cell viability assay for transfection and sensitivity of chemotherapeutic agents

After 24 h of transfection, the cells were passaged into 96-well plates (2000 cells/well) and allowed to proliferate. The absorbance reading was measured once per day for consecutive 6 days and calculated relative to day 1. In the detection of sensitivity to chemotherapeutic agents, cells were passaged into 96-well plates (5000 cells/well). After 24 h, cells were treated with various concentrations of temozolomide (TMZ) (SL PHARM, Beijing, China) and paclitaxel (PTX) (Beijing Union Pharmaceutical Factory, Beijing, China). The absorbance reading was measured after 48 h. Cell viability was determined by CellTiter-Glo Luminescent Cell Viability Assay (Promega, Madison, WI, USA) according to the manufacturer’s instruction. Data were presented as the means ± standard deviations from six repeated wells.

### Transwell cell migration and cell invasion assay

Falcon Cell Culture Inserts (BD Biosciences, Erembodegem, Belgium) and BD BioCoat Matrigel invasion chambers (BD Biosciences, Erembodegem, Belgium) were applied for cell migration and cell invasion assays, respectively. After 24 h for transfection, cells in the upper compartment of the chamber were suspended in serum-free medium and the bottom well contained medium supplemented with 15% fetal bovine serum. After incubation for 24 h (migration) or 48 h (invasion), cells on the upper side of the inserts were removed by cotton swabs, and cells on the underside were fixed and stained with crystal violet. Photos from three regions were taken from each insert, and then their numbers were calculated.

### Cell apoptosis and cell cycle assay

After transfection for 24 h, cell apoptosis was evaluated by staining with Annexin V and PI (Dojindo Molecular Technologies Inc., Shanghai, China) for 15 min at room temperature in the dark, followed by flow cytometry within 1 h (BD Biosciences, Erembodegem, Belgium). Cell apoptosis was analysed by Flowjo 7.6 software (TreeStar Inc., Ashland, OR, USA). After transfection for 48 h, cells were collected and fixed in 70% cold ethanol for at least 12 h at 4 °C. Cells were stained with 50 μg/ml propidium iodide (BD Biosciences, Erembodegem, Belgium) at room temperature for 30 min in the dark, and the cell cycle was analysed using the FACS Calibur system (BD Biosciences, Erembodegem, Belgium) and ModFit 4.0 software (Verity Software House, Topsham, ME, USA).

### Western blotting

After transfection for 48 h, total protein was extracted from cell pellets using RIPA buffer (TBS, 1% Nonidet P-40, 0.5% sodium deoxycholate, 0.1% SDS, 0.004% sodium azide) and quantified by the BCA Protein Assay Kit (Beyotime Biotechnology, Jiangsu, China). Then they were separated in 10% NuPAGE Bis-Tris SDS/PAGE Protein Gels (Invitrogen, Carlsbad, CA, USA) followed by transfer onto a polyvinylidene difluoride membrane (GE Healthcare, Piscataway, NJ). The membrane was blotted with primary antibodies followed by incubation with a secondary antibody (antibodies are shown in Additional file 2: Table S1). Proteins were visualized using ECL Plus Western Blotting Detection Reagents (GE Healthcare, Beijing, China).

### Vector construction and luciferase reporter assays

MiR-let-7b-5p and miR-let-7c-5p have the same potential binding site for MTDH and CALU, which was predicted by TargetScan (http://www.targetscan.org). Sequences of wild-type or mutant seed regions of MTDH and CALU were synthesized and cloned into the pMIR-REPORT luciferase vector (RiboBio, Guangzhou, China). Cells were seeded into 96-well plates, and 24 h after plating they were co-transfected with miR-let-7b-5p/miR-let-7c-5p or control, pMIR-REPORT vector, and pRL-TK vector using Lipofectamine 3000 (Invitrogen, Carlsbad, CA, USA). After transfection for 48 h, luciferase activity assays were then performed and normalized with Renilla activity according to the manufacturer’s protocols (Promega, Madison, WI, USA).

### In vivo tumourigenicity and metastasis assay

Female NOD/SCID (non-obese diabetic and severe combined immunodeficiency) mice (4–6 weeks old) (HFK Bio-Technology, Beijing, China) were kept under specific pathogen-free (SPF) conditions with a 12 h light/dark cycle and freely accessed autoclaved standard food and water. HMVII cells (5 × 10^6^) stably expressing miR-let-7b-5p, miR-let-7c-5p or negative control were suspended in 0.1 ml phosphate buffered saline and subcutaneously inoculated into the dorsal flank of NOD/SCID mice (*N* = 5 mice/group). Tumour sizes and animal weights were measured twice per week until animal sacrifice or the end of observation and tumour volumes were calculated using the following formula: V = (L × W^2^)/2 (V, volume; L, length; W, width of tumour). Stable miR-let-7b-5p, miR-let-7c-5p or negative control HMVII cells (1 × 10^6^) were injected through the tail vein into NOD/SCID mice (*N* = 5 mice/group). All mice were sacrificed 4 weeks after injection. The lungs and liver from each mouse were excised and embedded in paraffin for haematoxylin and eosin staining.

### Patient-derived xenograft models and treatment

Two mucosal melanoma tissues with different miR-let-7b-5p and miR-let-7c-5p expression were cut into ~ 100 mm^3^ fragments and then subcutaneously inoculated into NOD/SCID mice (*N* = 9 mice/group) to establish the PDX models. When tumours were approximately 150–200 mm^3^, mice were randomized into three subgroups (*N* = 3 mice/subgroup) and treated with saline, temozolomide (TMZ) (100 mg/m^2^, d1-d5/2 week, oral gavage), or paclitaxel (PTX) (10 mg/kg, twice weekly, intraperitoneal injection). The treatment lasted for 4 weeks. Tumour size and animal weights were measured twice per week until animal sacrifice or the end of observation, and tumour volumes were calculated using the following formula: Tumour growth inhibition (TGI) = 1 - (T_post_ - T_pre_) / (C_post_ - C_pre_) × 100% (T_pre_ = pre-drug tumour volume; T_post_ = post-drug tumour volume; C_pre_ = original tumour volume of the control group; C_post_ = final tumour volume of the control group; the same formula for volume as before).

### Statistical analysis

Statistical analysis was performed using SPSS20.0 for Windows software. Qualitative data were described as percentages. Quantitative data were described as means ± standard deviations. Pearson’s chi-square test, Fisher’s exact test and Mann-Whitney U test were used to test the correlation between qualitative variables such as the relationships between clinicopathological characteristics and miR-let-7b-5p / miR-let-7c-5p expression. Survival time including disease-free survival (DFS) and progression-free survival (PFS) used Kaplan-Meier and log-rank tests to analyse survival differences between groups. Univariate and multivariate cox regression analyses were employed to analyse predictive variety. Repeated measures analysis of variance was used to compare the difference in cell growth between two groups of cells. These tests were bilateral and the test level was defined as 0.05, such that *P* < 0.05 indicates statistically significant.

## Results

### Selection of cutoff values for expression of miR-let-7b and miR-let-7c

**Fig. 1:**
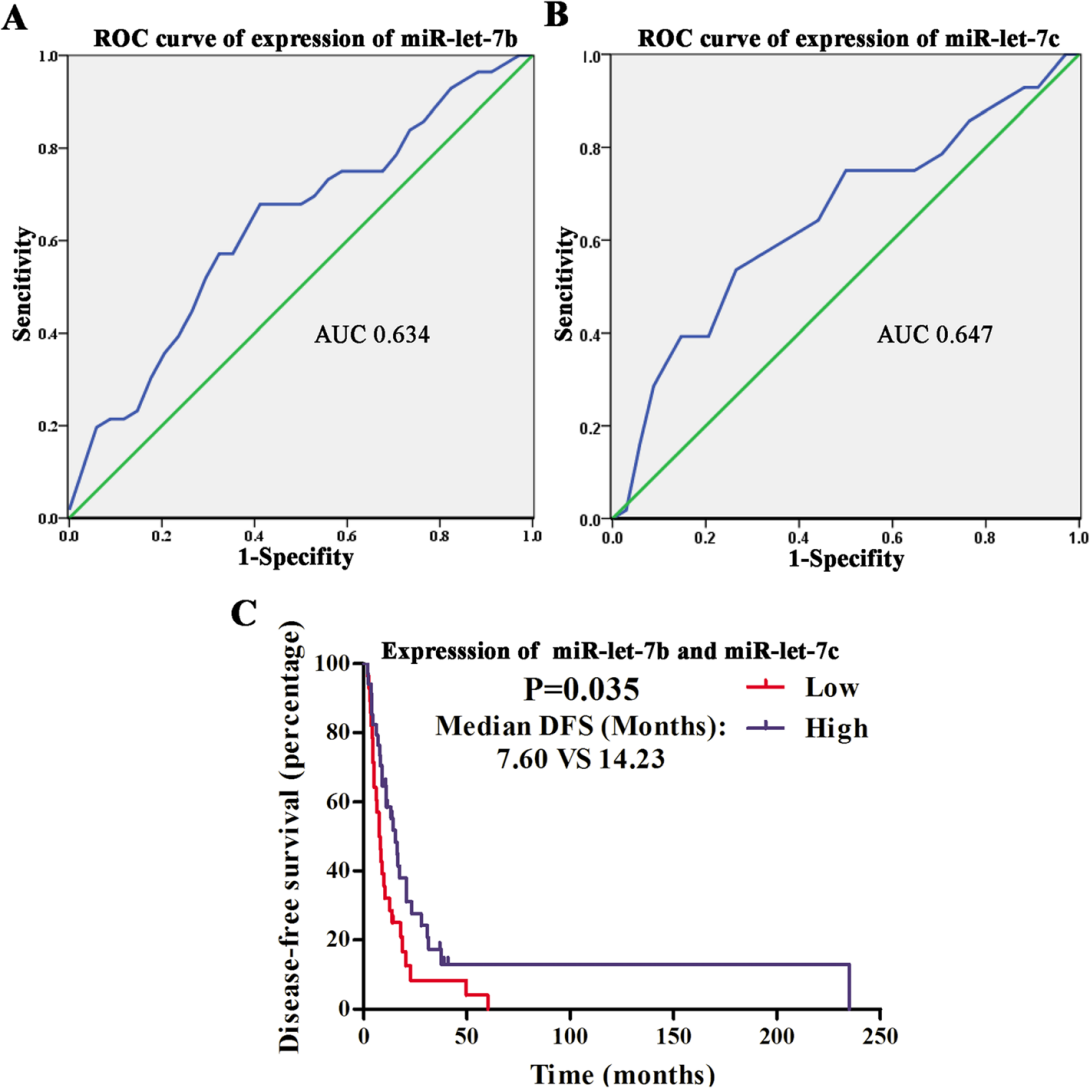
Cutoff scores of miR-let-7b and miR-let-7c and analysis of DFS in mucosal melanoma patients. Cutoff scores for miR-let-7b (**a**) and miR-let-7c (**b**) expression were 0.02452642 and 0.13255146, respectively. The AUC values for miR-let-7b (**a**) and miR-let-7c (**b**) expression were 0.634 and 0.647. **c** The median DFS stratified by expression of miR-let-7b versus miR-let-7c was 7.60 months versus 14.23 months (*P* = 0.035)

The expression of miR-let-7b and miR-let-7c decreased in 94 cases (88.7%) and in 89 cases (84.0%), respectively, of 106 mucosal melanoma patients compared with mucosal nevi. To further assess the correlations between miR-let-7b and miR-let-7c expression and clinical characteristics, the receiver operating characteristic (ROC) curve was used according to the median DFS in mucosal melanoma patients. The median DFS varied owing to different adjuvant therapy after surgery [15]. The cutoff scores for miR-let-7b and miR-let-7c expression were 0.02452642 and 0.13255146, respectively. To maximize the sum of sensitivity and specificity in the selection of cut off scores, the area under the ROC curve (AUC) values for miR-let-7b and miR-let-7c expressions were 0.634 (Fig. 1a) and 0.647 (Fig. 1b). Patients with expression both under the cutoff scores above were recorded as the low expression group and the others were the high expression group.

### Correlation between expression of miR-let-7b and miR-let-7c and clinical characteristics in mucosal melanoma patients

Among the 106 mucosal melanoma patients (52 males, 54 females) with a median age of 56 years (range 26–79 years), 46/106 (43.40%) were in the low expression group and 60/106 (56.60%) were in high expression group. The expression of miR-let-7b and miR-let-7c was correlated with Eastern Cooperative Oncology Group (ECOG) scores (*P* = 0.01). The percentages of mucosal melanoma patients with ECOG scores of 0, 1 and 2 were 33/93 (35.5%), 56/93 (60.2%) and 4/93 (4.3%), respectively. The proportion of high expression of miR-let-7b and miR-let-7c was higher for ECOG 0 than ECOG 1 and 2. There were no significant associations between expression of miR-let-7b and miR-let-7c and other clinical characteristics, such as gender, age, initial stage, metastasis, ulcer, lactate dehydrogenase (LDH) and gene mutations. The details of our findings are presented in Table 1.

**Table 1:** Correlations between the expression of miR-let-7b / miR-let-7c and patient characteristics

Clinical characteristics		Expression of miR-let-7b / miR-let-7c		*P* value
		Low (*n* = 46,%)	High (*n* = 60,%)	
Gender	Male	21 (40.4)	31 (56.6)	0.54
	Female	25 (46.3)	29 (53.7)	
Age	> 60	18 (40)	27 (60)	0.55
	≤60	28 (45.9)	33 (54.1)	
Initial stage	I + II	20 (46.5%)	23 (53.5%)	0.92
	III + IV	25 (45.5%)	30 (54.4%)	
Metastasis	With	24 (58.5)	17 (41.5)	0.11
(Liver/Brain)	Without	6 (35.3)	11 (64.7)	
Thickness	< 4 mm	1 (20.0)	4 (80.0)	0.91
	≥4 mm	9 (42.9)	12 (57.1)	
Ulcer	With	11 (28.9)	27 (71.1)	1.00
	Without	2 (25.0)	6 (75.0)	
LDH	< 240 IU/L	26 (45.6)	31 (54.4)	0.78
	≥240 IU/L	17 (48.6)	18 (51.4)	
ECOG	0	9 (27.3)	24 (72.7)	**0.01**
	1	32 (57.1)	24 (42.9)	
	2	2 (50)	2 (50)	
BRAF status	Wild type	43 (45.7)	51 (54.3)	0.17
	Mutant type	2 (25.0)	10 (75.0)	
CKIT status	Wild type	41 (42.3)	56 (57.7)	0.91
	Mutant type	5 (55.6)	4 (44.4)	
NRAS status	Wild type	32 (41.2)	45 (58.8)	0.44
	Mutant type	11 (52.4)	10 (47.6)	
PDGFR status	Wild type	43 (44.8)	53 (55.2)	1.00
	Mutant type	1 (50)	1 (50)	

*Initial stage* Stage of initial diagnosis, *LDH* Lactate dehydrogenase, *ECOG* Eastern Cooperative Oncology Group, *P* < 0.05 indicates statistically significant in bold

### Kaplan-Meier analysis and multivariate cox regression of clinical characteristics for DFS

As depicted in Fig. 1c and Additional file 2: Table S4, the median DFS calculated by Kaplan-Meier analysis for those with low versus high expression of miR-let-7b and miR-let-7c was 7.60 months (95%CI: 5.05–10.15) versus 14.23 months (95%CI: 7.84–20.62) (*P* = 0.035). In the Kaplan-Meier analysis for other clinical characteristics, DFS correlated with ulcer (*P* = 0.01) (Additional file 2: Table S4). The median DFS stratified by ulcer was 15.30 months (95%CI: 9.93–20.67) with ulcer versus 4.00 months (95%CI: 3.93–4.07) without ulcer (Additional file 2: Table S4). There were no significant correlations between DFS and other clinical characteristics, such as gender, age, initial stage, metastasis, LDH, ECOG and gene mutations (Additional file 2: Table S4). Ulcer (*P* = 0.009) and the expression of miR-let-7b and miR-let-7c (*P* = 0.03) were found to be independent predictors for mucosal melanoma recurrence after multivariate cox regression (Table 2).

**Table 2:** Multivariate cox regression analysis of potential predictors of DFS

Potential predictors		DFS		*P* value
		HR	95%CI	
Ulcer	Without / With	0.25	0.09–0.71	**0.009**
Expression of miR-let-7b / miR-let-7c	Low / High	0.43	0.20–0.92	**0.03**

*DFS* Disease-free survival, *CI* Confidence interval, *HR* Hazard ratio, *P* < 0.05 indicates statistically significant in bold

### miR-let-7b and miR-let-7c inhibited the growth of mucosal melanoma cell lines in vitro and in vivo

**Fig. 2:**
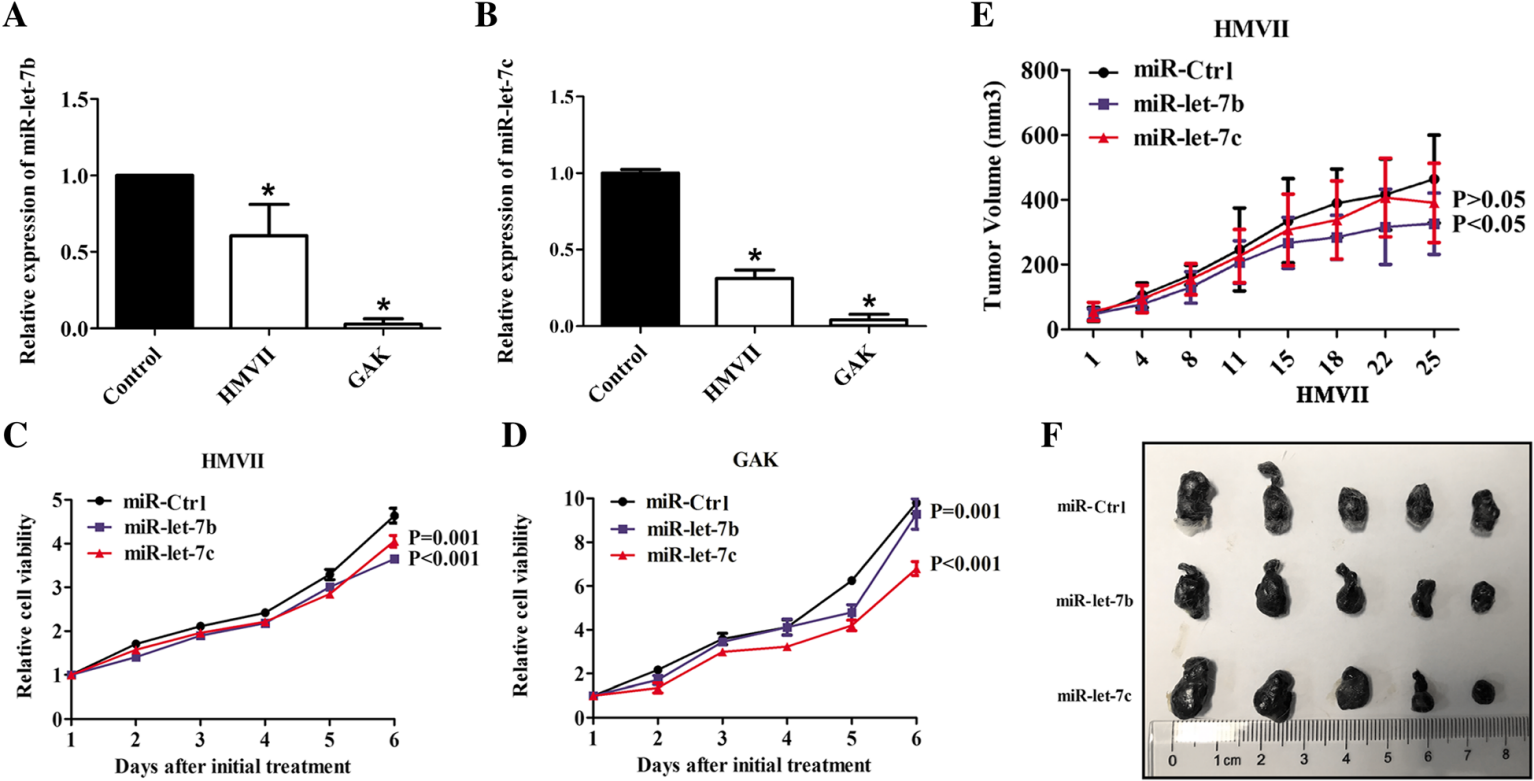
miR-let-7b and miR-let-7c inhibited growth of mucosal melanoma cells in vitro and in vivo. The expression of miR-let-7b (**a**) and miR-let-7c (**b**) was downregulated in mucosal melanoma cell lines. The expression was detected through qPCR with the internal control RNU6B in melanocytes. The results are presented as the means ± standard deviations from triplicate experiments and statistically analysed by Mann-Whitney U test. miR-let-7b and miR-let-7c inhibited the growth of HMVII (**c**) and GAK (**d**) in vitro. The cell viability was determined using the Cell Titer-Glo luminescent cell viability assay. **e** miR-let-7b and miR-let-7c inhibited the growth of HMVII in vivo. **f** Representative images of xenografts models stratified by miR-let-7b and miR-let-7c expressions. Lentiviral infection HMVII cells were subcutaneously inoculated into NOD/SCID mice. Each group contained 5 mice. The results are presented as the means ± standard deviations and statistically analysed by repeated measures analysis of variance. *P < 0.05

Downregulation of miR-let-7b and miR-let-7c in mucosal melanoma tissues and cell lines (Fig. 2a and b) suggested that miR-let-7b and miR-let-7c might act as tumour suppressors. To assess the biological function of miR-let-7b and miR-let-7c, transient transfection and virus infection were used to increase the expression level of miR-let-7b and miR-let-7c (Additional file 1: Figure S1). Cell viabilities were significantly suppressed in both HMVII (Fig. 2c) and GAK (Fig. 2d) cells with high expression of miR-let-7b or miR-let-7c in vitro (*P* ≤ 0.001). HMVII cells stably transfected with miR-let-7b, miR-let-7c or control were injected subcutaneously into NOD/SCID mice to establish tumourigenicity models in vivo. Compared with the control group, the inhibition rate of miR-let-7b was significantly inhibited (*P* < 0.05) (Fig. 2e and f).

### miR-let-7b and miR-let-7c inhibited migration and invasion in vitro and metastasis in vivo of mucosal melanoma cell lines

**Fig. 3:**
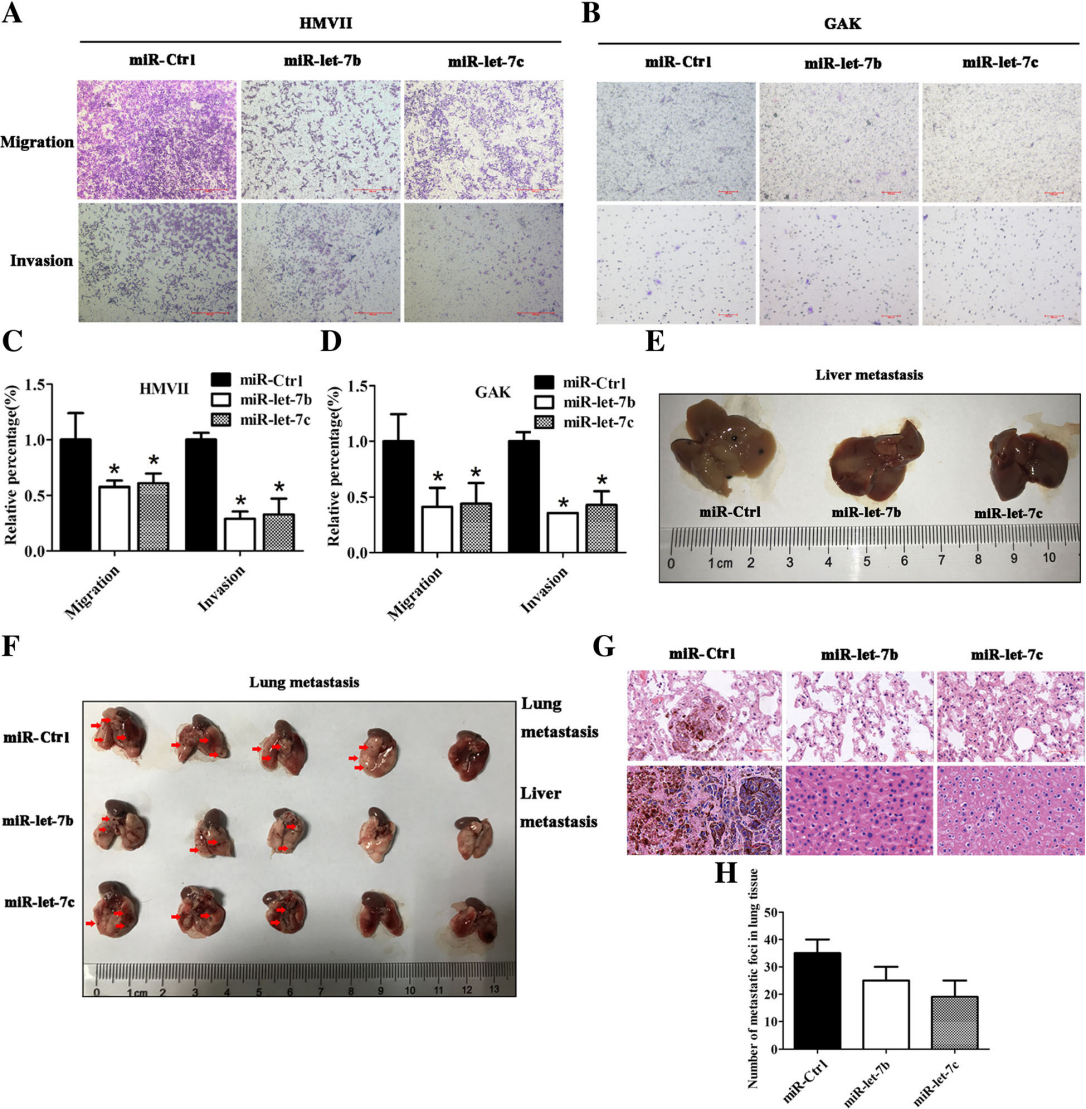
miR-let-7b and miR-let-7c inhibited mucosal melanoma cells in vitro migration, invasion and in vivo metastasis. miR-let-7b and miR-let-7c inhibited the migration and invasion of HMVII (**a**) and GAK (**b**) in vitro; statistical analyses are in (**c** and **d**). The results are presented as the means ± standard deviations from triplicate experiments and statistically analysed by Mann-Whitney U test. *P < 0.05. miR-let-7b and miR-let-7c inhibited liver (**e**) and lung (**f**) metastasis of HMVII. **g** Representative images of HE staining of liver and lung metastatic sections. **h** The number of metastatic foci in lung tissues is presented as the means ± standard deviations from triplicate counts

High expression of miR-let-7b and miR-let-7c predicted longer DFS in mucosal melanoma patients, indicating that miR-let-7b and miR-let-7c might play a role in inhibiting cell migration and invasion. Compared with control group, cell migration and invasion were inhibited for miR-let-7b (*P* < 0.05) and miR-let-7c (*P* < 0.05) in both HMVII and GAK cells (Fig. 3a-d). HMVII cells stably expressing miR-let-7b, miR-let-7c or negative control were injected through the tail vein into NOD/SCID mice. Only one mouse in the control group had metastatic foci in liver tissue. No such metastatic foci were observed in the miR-let-7b or miR-let-7c groups (Fig. 3e and g). The number of metastatic foci in lung tissue of 5 mice in the control group was more than that of the miR-let-7b or miR-let-7c group, as shown in Fig. 3f, g and h.

### miR-let-7b and miR-let-7c induced apoptosis and cell cycle arrest at G1 phase of mucosal melanoma cell lines

**Fig. 4:**
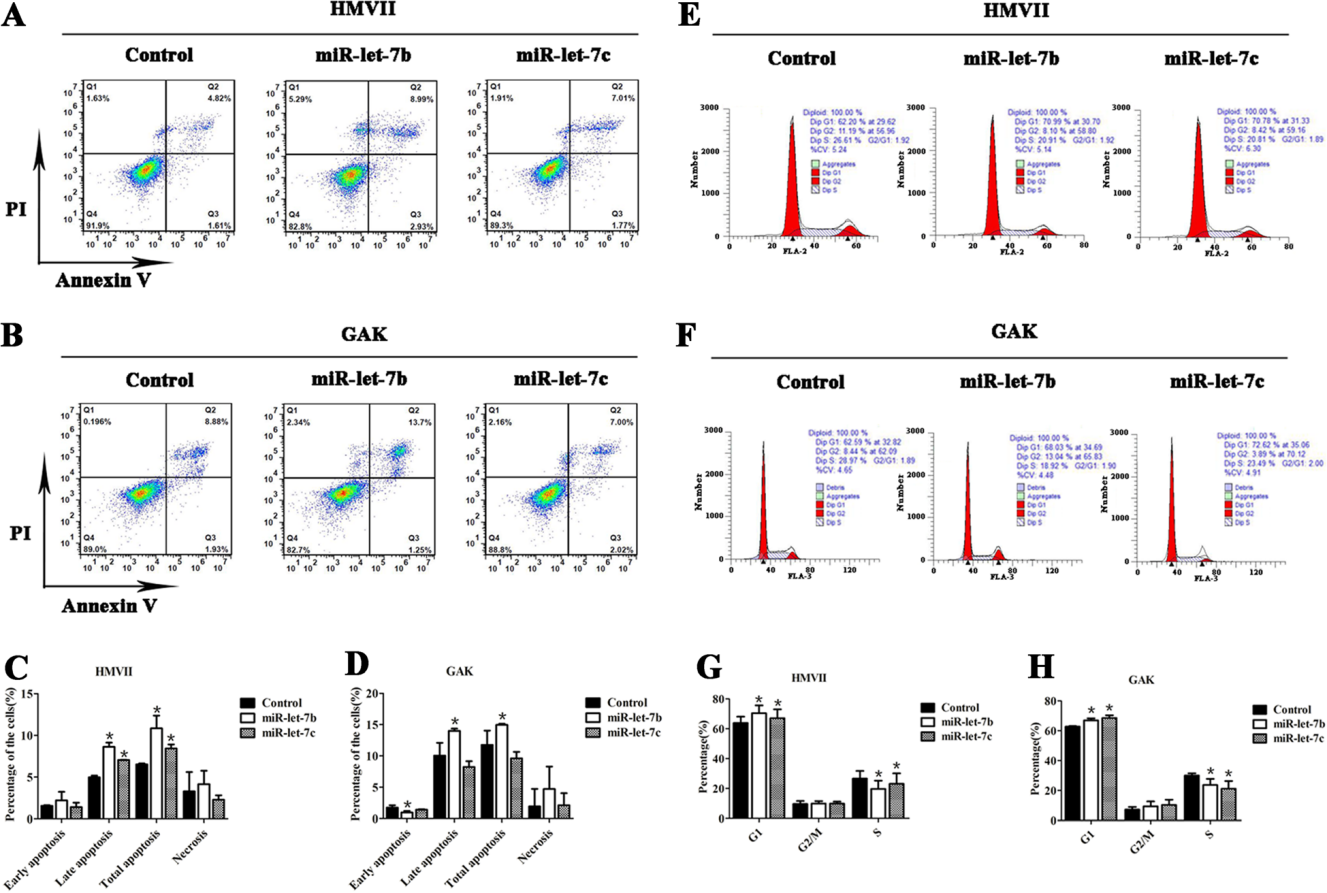
miR-let-7b and miR-let-7c induced mucosal melanoma cell apoptosis and cell cycle arrest at G1 phase. miR-let-7b and miR-let-7c induced late and total apoptosis of HMVII (**a**) cells; statistical analyses are in (**c**). miR-let-7b induced late and total apoptosis of GAK cells (**b**); statistical analyses are in (**d**). miR-let-7b and miR-let-7c induced apoptosis and cell cycle arrest at G1 phase of HMVII (**e**) and GAK (**f**) cells; analyses are in (**g** and **h**). The results are presented as the means ± standard deviations from triplicate experiments and statistically analysed by Mann-Whitney U test. *P < 0.05

In the detection of cell apoptosis, the percentage of late (*P* < 0.05) and total (*P* < 0.05) apoptotic cells in HMVII were significantly increased in the miR-let-7b and miR-let-7c groups compared with control (Fig. 4a and c). In the GAK cell line, higher percentages of late (*P* < 0.05) and total (*P* < 0.05) apoptotic cells were observed in the miR-let-7b group (*P* < 0.05), but this was not observed in the miR-let-7c group (Fig. 4b and d). The result showed that a significantly increased proportion of cells arrested in G1 phase (*P* < 0.05) and a downregulated proportion of cells in S phase (*P* < 0.05) in the miR-let-7b and miR-let-7c groups compared with the control group in HMVII and GAK cells (Fig. 4e-h).

### miR-let-7b and miR-let-7c inhibited the expression of metadherin (MTDH) and calumenin (CALU) via binding to their 3′ UTR

**Fig. 5:**
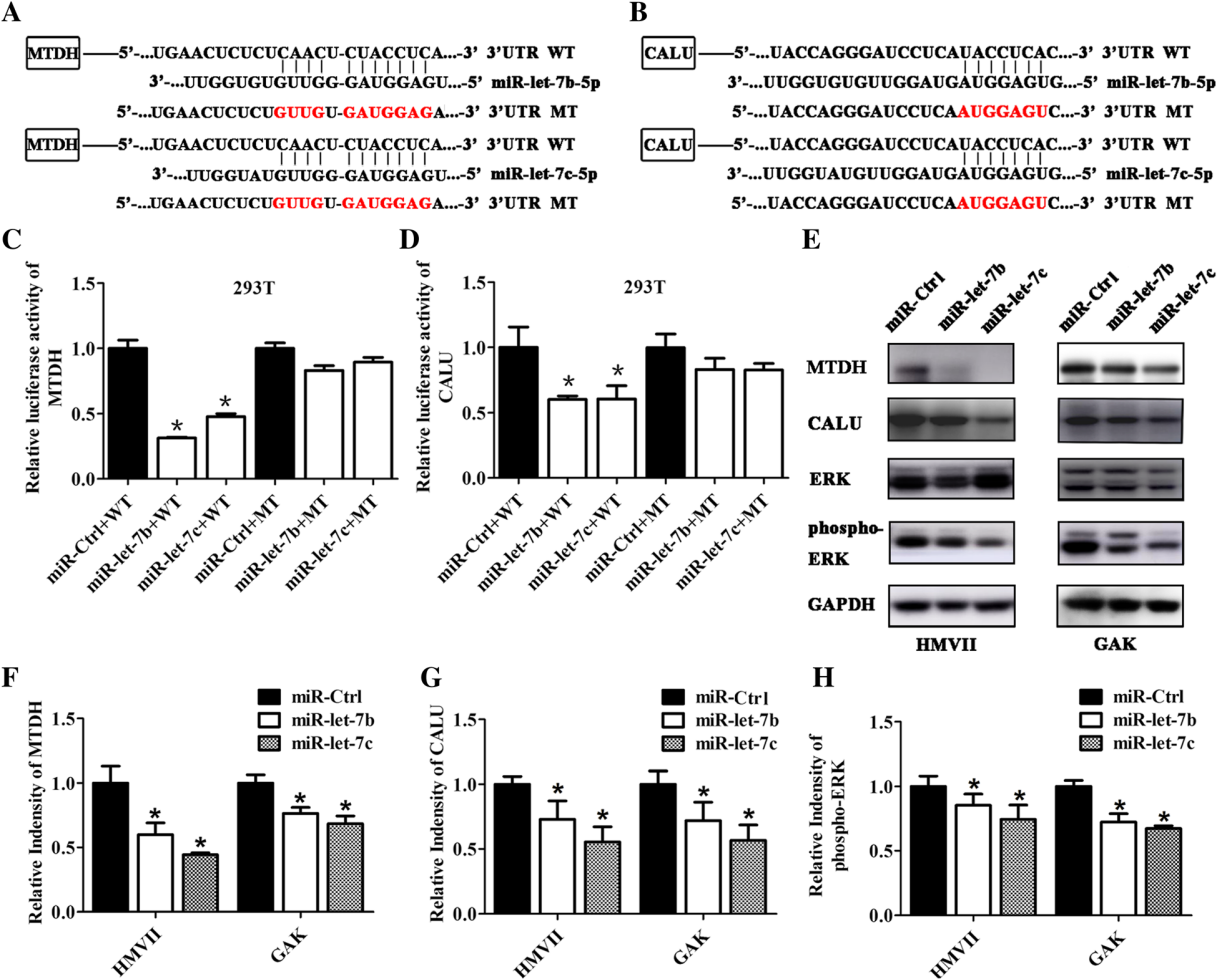
miR-let-7b and miR-let-7c inhibited MTDH and CALU via binding to their 3′ UTRs. The sequences of miR-let-7b, miR-let-7c and potential binding sites in the 3′ UTRs of target genes MTDH (**a**) and CALU (**b**) were predicted by software packages. miR-let-7b and miR-let-7c inhibited the luciferase activity of wild-type MTDH (**c**) and CALU (**d**) 3′ UTR in 293 T cell lines. The results are presented as the means ± standard deviations from six replicates and statistically analysed by Mann-Whitney U test. **e** Western blotting was used to detect proteins influenced by miR-let-7b and miR-let-7c; GAPDH is shown as a loading control. The relative intensity of MTDH (**f**), CALU (**g**) and phospho-ERK (**h**) influenced by miR-let-7b and miR-let-7c were quantified by Image J software and analysed statistically. The relative intensity of MTDH and CALU were normalized to GAPDH, and phosphorylated bands were normalized to total bands. The results are presented as the means ± standard deviations from triplicate scans and statistically analysed by Mann-Whitney U test. *P < 0.05

miRNAs exert their effect through inhibition of target genes. Therefore, it is important to clarify the mechanisms of miRNAs via identifying direct target genes. Three mucosal nevi as negative control and 23 mucosal melanoma tissues with low expression of miR-let-7b and miR-let-7c selected from 106 mucosal melanoma tissues were used to detect mRNA level. A total of 9 genes were upregulated among mucosal melanoma: MTDH, CALU, SULF1, FBXO32, BZW2, ABL2, PLD3, MLXIP and CERS2. According to the prediction software packages: miRNA, TargetScan, and DIANA-microT, 2 genes, MTDH and CALU were predicted target genes of miR-let-7b and miR-let-7c, with higher probability of preferential conservation (Table 3). The sequences of miR-let-7b and miR-let-7c and their potential binding sites in the 3′ UTR of predicted target genes MTDH and CALU are shown in Fig. 5a and b. The results from luciferase reporter assays revealed that expression of miR-let-7b and miR-let-7c inhibited luciferase activity of wild-type MTDH (*P* < 0.05) and CALU (*P* < 0.05) but had no effect on mutant MTDH and CALU in the 293 T cell line (Fig. 5c and d). Thus, it was confirmed that MTDH and CALU were direct targets of miR-let-7b and miR-let-7c. Downregulated MTDH and CALU protein levels were observed in HMVII and GAK cells with increased miR-let-7b and miR-let-7c levels (*P* < 0.05) (Fig. 5e-g). The RAS/RAF/MEK/ERK pathway is a prime therapeutic target in melanoma, and the protein level of phospho-ERK was significantly down-regulated with high expression of miR-let-7b and miR-let-7c in HMVII and GAK cells (*P* < 0.05) (Fig. 5e and h).

**Table 3:** Upregulated genes in mucosal melanoma tissues were predicted by prediction software packages as potential target genes

Genes	Fold change	TargetScan	miRanda	DIANA-microT
MTDH	4.01	Include	Include	Include
CALU	3.32	Include	Include	Include
SULF1	5.02	Include	Exclude	Include
FBXO32	8.03	Exclude	Exclude	Include
BZW2	3.99	Exclude	Include	Exclude
ABL2	2.15	Exclude	Exclude	Include
PLD3	4.11	Exclude	Include	Exclude
MLXIP	2.05	Exclude	Include	Exclude
CERS2	2.45	Exclude	Exclude	Exclude

Include: Prediction software package included the gene as a target of miR-let-7b and miR-let-7c. Exclude: Prediction software package excluded the gene as a target of miR-let-7b and miR-let-7c. Fold change: the mRNA expression level compared with negative control. Nine genes were upregulated among mucosal melanoma. Among the prediction software packages: miRNA, TargetScan, and DIANA-microT, 2 genes were predicted as targets of miR-let-7b and miR-let-7c with higher probability of preferential conservation. Although SULF1 was predicted as a target gene by TargetScan, its site had lower probability of preferential conservation

### MTDH and CALU partially reversed the function of miR-let-7b and miR-let-7c in vitro

**Fig. 6:**
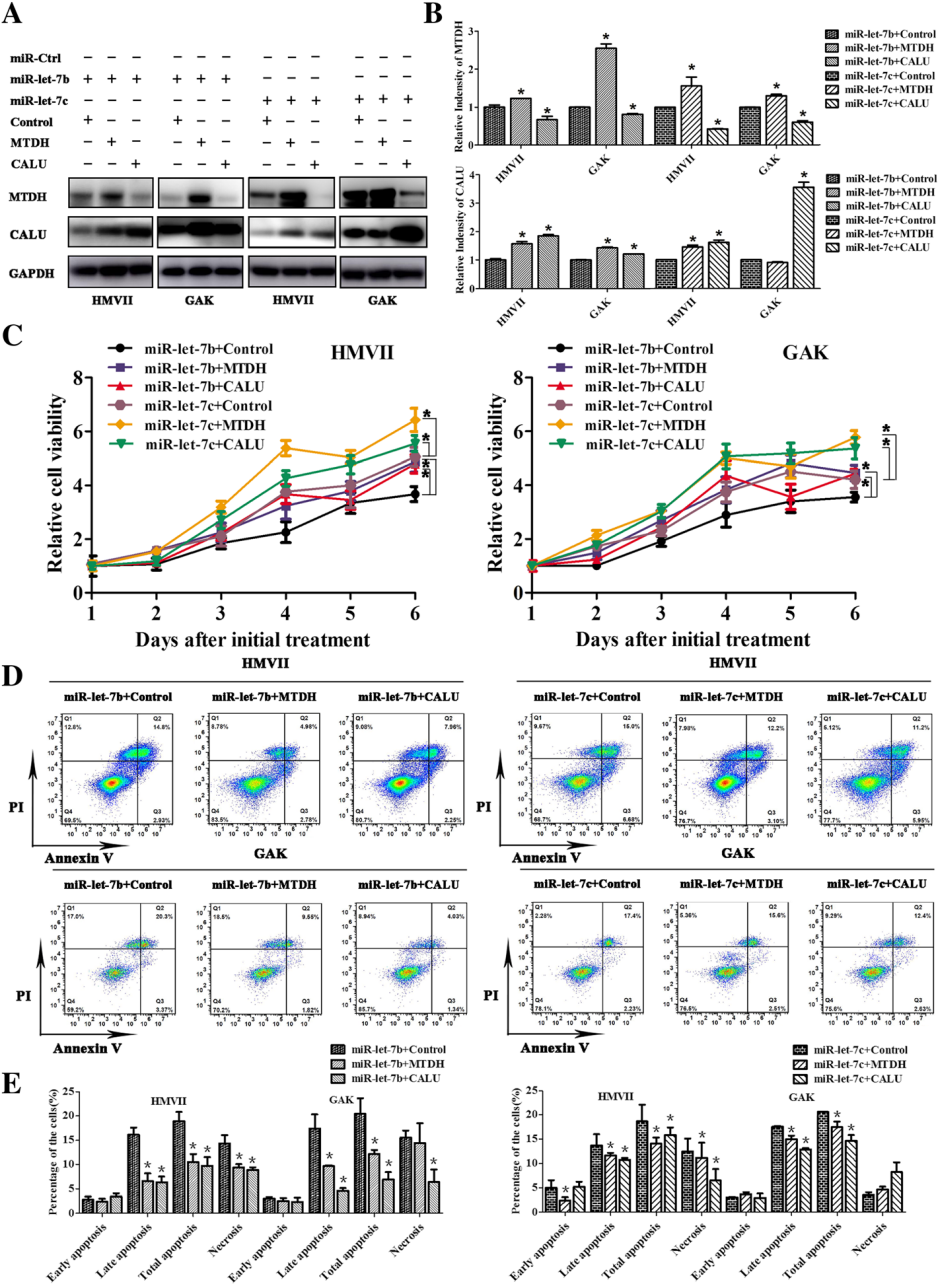
MTDH and CALU partially reversed the function of miR-let-7b and miR-let-7c in vitro. **a** Western blotting was used to detect MTDH and CALU influenced by co-transfection of miR-let-7b/miR-let-7c and MTDH/CALU in HMVII and GAK; GAPDH is shown as a loading control. **b** The relative intensity of MTDH and CALU after co-transfection was quantified by Image J software and analysed statistically. The relative intensity of MTDH and CALU were normalized to GAPDH. The results are presented as the means ± standard deviations from triplicate scans and statistically analysed by Mann-Whitney U test. MTDH and CALU reversed cell growth (**c**) and apoptosis (**d** and **e**) in response to miR-let-7b and miR-let-7c in HMVII and GAK cells. The cell viability was determined using the Cell Titer-Glo luminescent cell viability assay. The results are presented as the means ± standard deviations and statistically analysed by repeated measures analysis of variance. The cell apoptosis (**e**) results are presented as the means ± standard deviations of triplicate experiments and statistically analysed by Mann-Whitney U test. *P < 0.05

With the identification of the direct target genes MTDH and CALU, rescue experiments were performed to test the interaction of miR-let-7b/miR-let-7c and MTDH/CALU in HMVII and GAK cells. Compared with control, the protein level of MTDH (*P* < 0.05) increased after co-transfection with MTDH (Fig. 6a-b), and CALU (*P* < 0.05) was increased after co-transfection with CALU (Fig. 6a-b). Furthermore, compared with transfected miR-let-7b or miR-let-7c alone, cell growth (*P* < 0.05) and apoptosis (*P* < 0.05) were reversed with co-transfection of MTDH and CALU (Fig. 6c-e). Thus, MTDH and CALU partially reversed function of miR-let-7b and miR-let-7c in vitro.

### Survival analysis between expression of miR-let-7b and miR-let-7c and PFS with temozolomide-based or paclitaxel-based chemotherapy

**Fig. 7:**
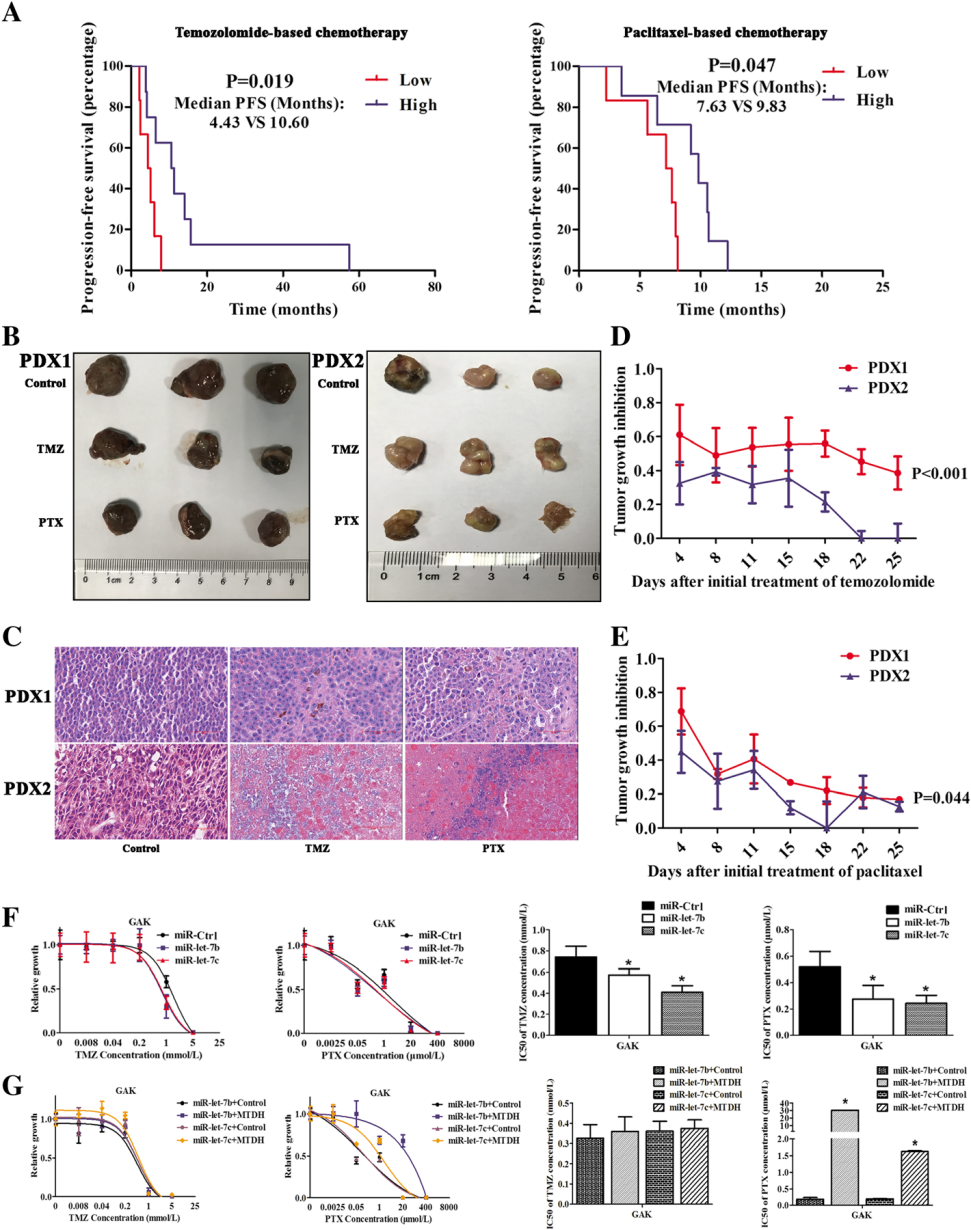
miR-let-7b and miR-let-7c increased the sensitivity to temozolomide and paclitaxel in mucosal melanoma. **a** miR-let-7b and miR-let-7c prolonged PFS with temozolomide-based and paclitaxel-based chemotherapy in mucosal melanoma patients. Data were calculated using Kaplan-Meier analysis. Representative images for PDX models of tumour volume (**b**) and HE staining (**c**) treated with TMZ and PTX. The tumour growth inhibition (TGI) was significantly higher in PDX1 than in PDX2 (*P* < 0.001 for treatment of TMZ and *P* = 0.044 for treatment of PTX) (**d** and **e**). Each subgroup contained 3 mice. The results are presented as the means ± standard deviations and statistically analysed by repeated measures analysis of variance. **f** miR-let-7b and miR-let-7c could increase sensitivity of TMZ and PTX treatment in GAK. **g** MTDH reversed sensitivity to PTX for miR-let-7b and miR-let-7c in vitro. The cell viability was detected 48 h after treatment in different concentrations of chemotherapeutic agents using the Cell Titer-Glo luminescent cell viability assay. The results are presented as the means ± standard deviations from six replicates and statistically analyzed by Mann-Whitney U test. *P < 0.05

As shown in Fig. 7a and Additional file 2: Table S5, the median PFS with temozolomide-based chemotherapy calculated by Kaplan-Meier analysis, for subjects with low versus high expression of miR-let-7b and miR-let-7c was 4.43 months (95%CI: 1.19–7.67) versus 10.60 months (95%CI: 3.86–17.35) (*P* = 0.02). For paclitaxel-based chemotherapy, the median PFS for the low versus high expression of miR-let-7b and miR-let-7c groups was 7.63 (95%CI: 6.43–8.31) versus 9.83 months (95%CI: 4.87–14.79) (*P* = 0.047) (Fig. 7a and Additional file 2: Table S6). After Kaplan-Meier analysis for other clinical characteristics, PFS with temozolomide-based chemotherapy only correlated with the expression of miR-let-7b and miR-let-7c (*P* = 0.02) (Additional file 2: Table S5). The median PFS with paclitaxel-based chemotherapy also correlated with metastasis (*P* = 0.045) and NRAS status (*P* = 0.049) (Additional file 2: Table S6), but no significance from further multivariate cox regression was observed (Additional file 2: Table S7).

### Sensitivity of temozolomide and paclitaxel in PDX models and mucosal melanoma cell lines

In PDX models, PDX1 and PDX2 models with high and low expression of miR-let-7b and miR-let-7c were separately selected in four established PDX models of mucosal melanoma patients (Additional file 2: Table S3). The results demonstrated that tumour growth inhibition (TGI) was significantly higher in PDX1 than in PDX2 (*P* < 0.001 for treatment of TMZ and *P* = 0.044 for treatment of PTX) (Fig. 7b-e). In addition, miR-let-7b and miR-let-7c only increased the sensitivity of TMZ and PTX treatment in GAK cells (*P* < 0.05), but not in HMVII cells (Fig. 7f). Therefore, certain mucosal melanoma cells with high expression of miR-let-7b and miR-let-7c may be sensitive to these two chemotherapeutic agents. The sensitivity to PTX could be reversed by MTDH (*P* < 0.05) in vitro (Fig. 7g). This finding could further confirm the effect of MTDH as a direct target gene of miR-let-7b and miR-let-7c.

## Discussion

In our study, miR-let-7b and miR-let-7c were downregulated in most mucosal melanoma tissues compared with mucosal nevi. However, the expression of miR-let-7 family members varied in melanoma and other cancer types. Let-7a, let-7b, let-7d, let-7e and let-7g were decreased, while let-7i was increased in melanoma [14]. Most members of the miR-let-7 family were down-regulated in lung, colon, breast, oesophageal and endometrial cancer tissues [16–20], while let-7b and let-7i were upregulated in lymphoma [21]. The initial studies of tumourigenicity and metastasis were mainly based on miRNA expression profiling methods from tumour tissues [16] or data from cell lines [22]. To further evaluate miR-let-7 as a potential diagnostic and prognostic biomarker, clinical characteristics of mucosal melanoma patients were analysed in our study. For tumour patients, ECOG score was associated with tumour burden. Thus, the correlation between the expression of miR-let-7b and miR-let-7c with ECOG could predict progression and metastasis of mucosal melanoma patients. The expression of miR-let-7b and miR-let-7c was related to recurrence for mucosal melanoma patients using Kaplan-Meier analysis and multivariate cox regression. Ulcer was another independent predictor of mucosal melanoma recurrence, but ulcer indicated longer DFS in our study which was opposite to the concept of risk factors for early-stage melanoma according to NCCN guidelines [23]. Because primary mucosal melanomas are located in tracts of the body, it is difficult to detect this disease. The time for diagnosis is later for cutaneous melanoma, and the proportion of ulcers would be increased in mucosal melanoma patients. The correlation of ulcer and DFS stratified by different stage remains to be further confirmed.

MiRNAs can mediate multiple target gene regulation. Thus, it is essential to further validate their potential functions using cell experiments in vitro and in vivo. In previous reports that miR-let-7a and miR-let-7b led to obvious reduction of melanoma cell growth and proliferation in soft agar assays [24], reduction of cell migration and invasion [22, 25] and also increased numbers of cells in G1 phase [24]. miR-let-7b and miR-let-7c had the same effect on proliferation, migration, invasion and cell cycle in mucosal melanoma as in cutaneous melanoma. Our research further confirmed the inhibitory function of subcutaneous tumourigenicity and metastasis of lung and liver for miR-let-7b and miR-let-7c in vivo. Of note, the cell apoptosis results were newly shown in melanoma. The percentages of late and total apoptotic cells were significantly increased with miR-let-7b and miR-let-7c, except miR-let-7c in GAK cells. The in vitro and vivo experiments offered more details to support clinical data for mucosal melanoma progression and recurrence.

As the prediction software suggested, MTDH and CALU were potential targets for miR-let-7b and miR-let-7c, and these were also confirmed by luciferase reporter assay in our study. MTDH was reported as an oncogene in several cancer types, such as glioma, neuroblastoma, breast cancer, prostate cancer, liver and oesophagus cancer, especially mucosal melanoma [26]. MTDH exerted its function by stimulating proliferation, invasion, cell survival and chemo-resistance [26]. AEG-1/MTDH/LYRIC was found to be involved in multiple signalling pathways, such as PI3K/AKT, NF-κB, Wnt/β-catenin and MAPK [26]. Another study suggested that AEG-1/MTDH/LYRIC participated in transforming growth factor (TGF) β1-induced epithelial-mesenchymal transition through activation of the p38 MAPK pathway in proximal tubular epithelial cells [27]. Our study confirmed that MTDH increased the expression of GDF-15 (Additional file 1: Figure S2), which is also a member of the TGF-β pathway and further explained the interaction between MTDH and TGF-β. The expression of CALU differed from cancer types and was downregulated in head and neck squamous cell carcinoma [28] and lung squamous cell carcinoma [29], among others [30, 31]. CALU expression was found to be increased in mucosal melanoma tissues through microarray hybridization and gene expression analyses and immunohistochemistry (Additional file 1: Figure S3). The outcome was in concordance with that in colon cancer [32]. Distinct from the extracellular effect of CALU-1/− 2 in downregulated cancers [31], CALU-15 facilitated the nuclear expression of nucleus GDF-15, which then mainly promoted cell migration and tumour metastasis [33]; our study confirmed that CALU promoted the expression of GDF-15 (Additional file 1: Figure S2). In CALU downregulated cancer types, the CALU-1/− 2 / fibulin-1 complex bound to fibronectin and inhibited ERK-1/2 signalling and cell migration in an integrin- and syndecan-dependent manner [31]. In our study, it was observed that CALU, most likely CALU-15, promoted malignant progression of mucosal melanoma.

The miR-let-7 family also focuses on the CDK pathway and RAS/RAF/MEK/ERK pathway. On one hand, it was previously reported that miR-let-7b repressed expression of cyclins D1 (CCND1), D3, CDK4, and cyclin A [24]. CCND1 was shown to be a potential target of the miR-let-7 family through binding to its 3′ UTR in vitro luciferase assays [24]. This mechanism was paralleled by cell cycle arrest at G1 phase, which was also confirmed by our research. On the other hand, our study revealed that phospho-ERK was downregulated with high expression of miR-let-7b and miR-let-7c in HMVII and GAK cells.

Chemotherapy is a traditional treatment of advanced melanoma. In the present study, PFS was significantly longer in mucosal melanoma patients with high expression of miR-let-7b and miR-let-7c for temozolomide-based and paclitaxel-based chemotherapy. The effects of TMZ and PTX effect in PDX models with different expression levels of miR-let-7b and miR-let-7c supported the hypothesis that miR-let-7 family enhanced sensitivity to temozolomide-based and paclitaxel-based chemotherapy. In mucosal melanoma cell lines, miR-let-7b and miR-let-7c only increased the sensitivity to TMZ and PTX treatment in GAK, but not in HMVII cells. This finding suggests that certain mucosal melanoma cells with high expression of miR-let-7b and miR-let-7c would have increased sensitivity to these two chemotherapeutic agents. Due to the lack of mucosal melanoma cell lines, the results remained to be further confirmed in other cell lines. There are few reports related to TMZ in other cancers. An initial study showed that let-7e was downregulated in ovarian cancer cells, which are resistant to paclitaxel [34]. miR-200c could increase sensitivity of gastric cancer cells to paclitaxel [35]. In addition, temozolomide-based and paclitaxel-based chemotherapy can be combined treatment with cisplatin or carboplatin. As previous reports indicated that the miR-let-7 family enhanced the sensitivity of various cancer types to cisplatin [19, 36], it may also have an effect on mucosal melanoma. However, we did not detect the effect of cisplatin in our study. The results from our study suggested that MTDH reversed sensitivity to PTX for miR-let-7b and miR-let-7c in vitro. In previous studies, AEG-1/MTDH/LYRIC led to drug resistance to chemotherapeutic agents, including 5-fluorouracil (5-FU), doxorubicin, paclitaxel, and cisplatin, as well as to targeted therapies [37]. The function of drug resistance in AEG-1/MTDH/LYRIC was not only involved in multidrug resistance gene (MDR) 1, H-RAS, myc, NFκB and PI3K/AKT but also exerted a novel function as an RNA binding protein and microRNA-directed gene silencing via an interaction with staphylococcal nuclease and tudor domain containing 1 (SND1) [37].

Our study was carried out on a small population. In the future, the correlation between expression of miR-let-7b and miR-let-7c with recurrence and benefit of chemotherapy will be validated in a larger cohort. Because of the lack of mucosal melanoma cell lines and PDX models, cell lines and PDX models originating from various sites would be included for further research. In addition, our study is a retrospective project and we hope to perform further prospective studies in the future.

## Conclusion

In conclusion, our results suggested that miR-let-7b and miR-let-7c inhibited the recurrence of mucosal melanoma through inhibiting cell growth, migration and invasion, inducing cell apoptosis and cell cycle arrest by targeting MTDH and CALU. In addition, miR-let-7b and miR-let-7c increased the sensitivity to chemotherapeutic agents by targeting MTDH.

## Additional files

Additional file 1: **Figure S1.** The increased expression of miR-let-7b and miR-let-7c in mucosal melanoma cells. The expression of miR-let-7b (A) and miR-let-7c (B) was upregulated in HMVII cells through transient transfection. The expression of miR-let-7b (C) and miR-let-7c (D) was upregulated in GAK cells through transient transfection. The expression of miR-let-7b (E) and miR-let-7c (F) was upregulated in HMVII cells through virus infection. The expression was detected through qPCR with an internal control RNU6B in melanocytes. The results are presented as the means ± standard deviations from triplicate experiments and statistically analysed by Mann-Whitney U test. *P < 0.05. **Figure S2.** The detection of GDF-15 in mucosal melanoma cells. (A) Western blotting was used to detect GDF-15. GAPDH is shown as a loading control. (B) The relative intensity of GDF-15 influenced by miR-let-7b, miR-let-7c and co-transfection with MDTH or CALU was quantified by software Image J software and analysed statistically. The relative intensity of GDF-15 was normalized to GAPDH. The results are presented as the means ± standard deviations from triplicate scans and statistically analysed by Mann-Whitney U test. *P < 0.05. **Figure S3.** Immunohistochemistry for CALU in mucosal melanoma tissues. CALU protein from mucosa, mucosal nevi and mucosal melanoma patients was stained by immunohistochemistry. CALU protein staining was strongly positive in mucosal melanoma, weakly positive in mucosal nevi and negative in mucosal tissue. (ZIP 2193 kb)

Additional file 2: **Table S1.** Antibodies used in this study. **Table S2.** Origins and genetic aberrations of mucosal melanoma cells used in this study. **Table S3.** Clinical characteristics of established PDX models from mucosal melanoma patients used in this study. **Table S4.** Kaplan-Meier analysis of clinical characteristics of DFS. **Table S5.** Kaplan-Meier analysis of clinical characteristics of PFS in temozolomide-based chemotherapy. **Table S6.** Kaplan-Meier analysis of clinical characteristics of PFS in paclitaxel-based chemotherapy. **Table S7.** Multivariate cox regression analysis of potential predictors of PFS in paclitaxel-based chemotherapy. (ZIP 112 kb)

## Abbreviations

AUC: Area under the curve
CALU: Calumenin
CCND1: Cyclin D1
DFS: Disease-free survival
ECOG: Eastern Cooperative Oncology Group
LDH: Lactate dehydrogenase
miRNA: microRNA
MTDH: Metadherin
NOD/SCID: Non-obese diabetic and severe combined immunodeficiency
PDX: Patient-derived xenograft
PFS: Progression-free survival
PTX: Paclitaxel
qPCR: Quantitative polymerase chain reaction
ROC: Receiver operating characteristic curve
SPF: Specific pathogen-free
TGI: Tumour growth inhibition
TMZ: Temozolomide
