# Supplementary figures and images for: miR-let-7b and miR-let-7c suppress tumourigenesis of human mucosal melanoma and enhance the sensitivity to chemotherapy
Source: J Exp Clin Cancer Res. 2019 May 22;38:212. doi: 10.1186/s13046-019-1190-3 (PMC6532197; doi:10.1186/s13046-019-1190-3)

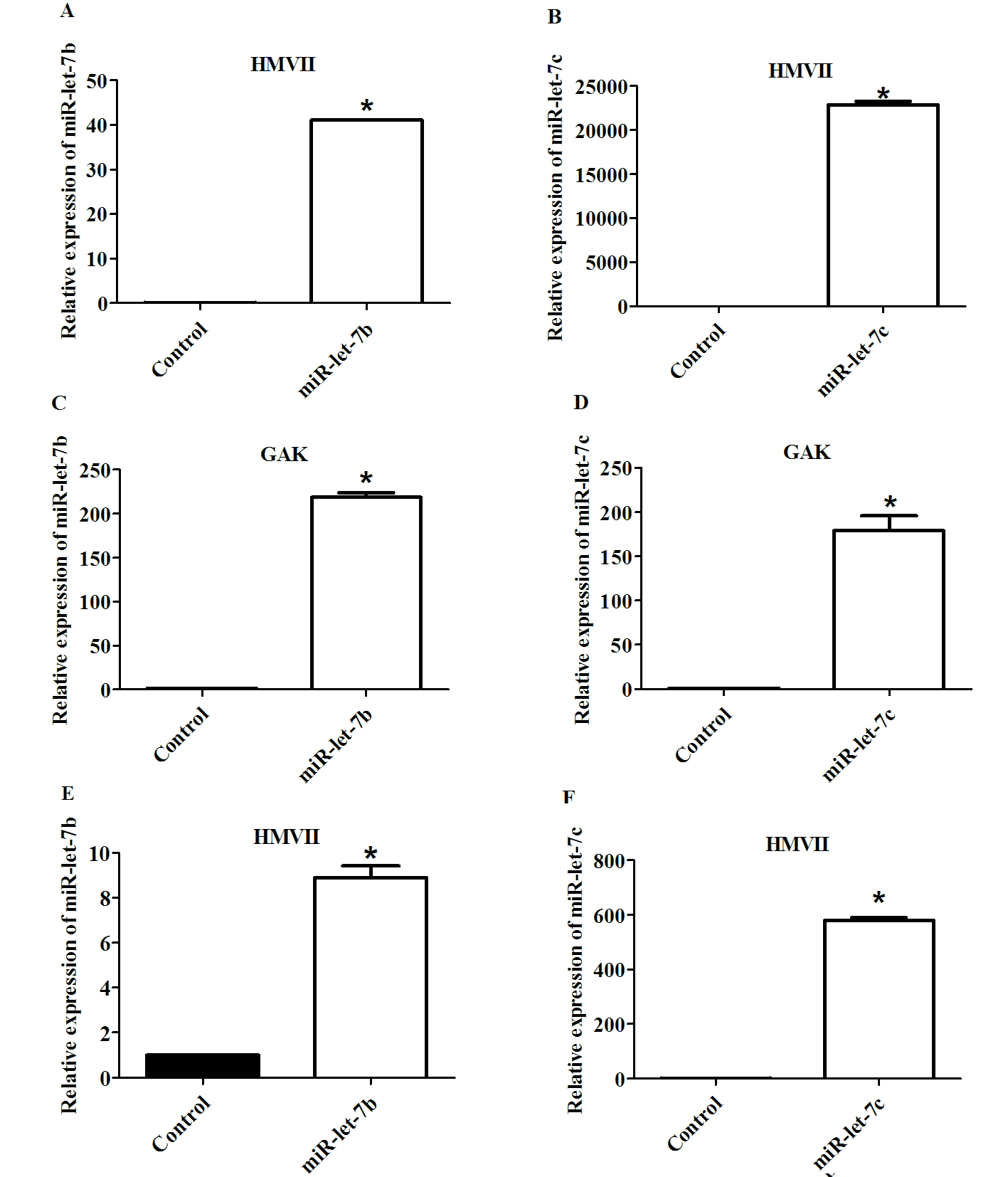

Supplement: Supplementary file 1 — Figure S1. The increased expression of miR-let-7b and miR-let-7c in mucosal melanoma cells. The expression of miR-let-7b (A) and miR-let-7c (B) was upregulated in HMVII cells through transient transfection. The expression of miR-let-7b (C) and miR-let-7c (D) was upregulated in GAK cells through transient transfection. The expression of miR-let-7b (E) and miR-let-7c (F) was upregulated in HMVII cells through virus infection. The expression was detected through qPCR with an internal control RNU6B in melanocytes. The results are presented as the means ± standard deviations from triplicate experiments and statistically analysed by Mann-Whitney U test. *P < 0.05. Figure S2. The detection of GDF-15 in mucosal melanoma cells. (A) Western blotting was used to detect GDF-15. GAPDH is shown as a loading control. (B) The relative intensity of GDF-15 influenced by miR-let-7b, miR-let-7c and co-transfection with MDTH or CALU was quantified by software Image J software and analysed statistically. The relative intensity of GDF-15 was normalized to GAPDH. The results are presented as the means ± standard deviations from triplicate scans and statistically analysed by Mann-Whitney U test. *P < 0.05. Figure S3. Immunohistochemistry for CALU in mucosal melanoma tissues. CALU protein from mucosa, mucosal nevi and mucosal melanoma patients was stained by immunohistochemistry. CALU protein staining was strongly positive in mucosal melanoma, weakly positive in mucosal nevi and negative in mucosal tissue. (ZIP 2193 kb) [file 13046_2019_1190_MOESM1_ESM.zip › supplement figure1-new.tif]

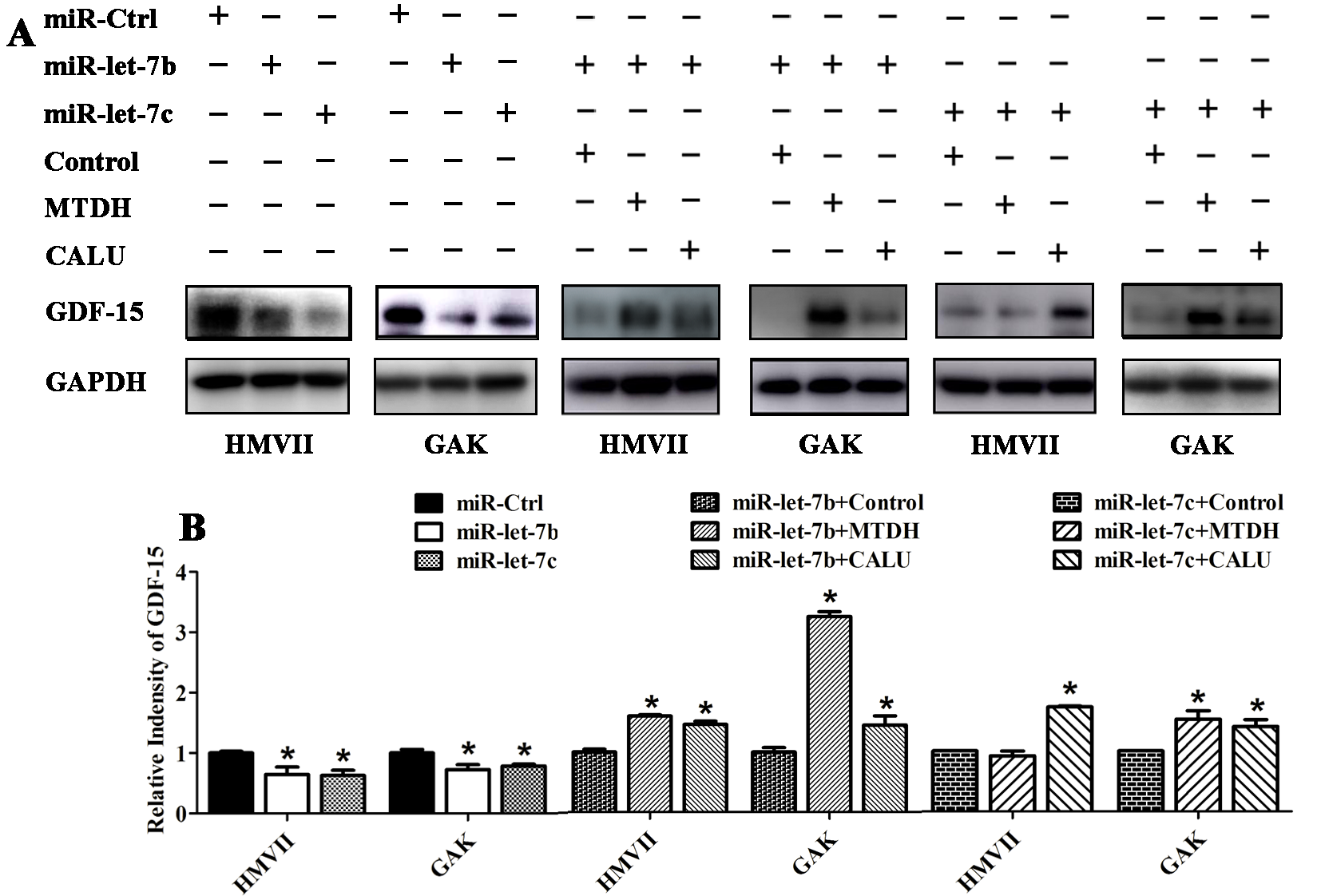

Supplement: Supplementary file 1 — Figure S1. The increased expression of miR-let-7b and miR-let-7c in mucosal melanoma cells. The expression of miR-let-7b (A) and miR-let-7c (B) was upregulated in HMVII cells through transient transfection. The expression of miR-let-7b (C) and miR-let-7c (D) was upregulated in GAK cells through transient transfection. The expression of miR-let-7b (E) and miR-let-7c (F) was upregulated in HMVII cells through virus infection. The expression was detected through qPCR with an internal control RNU6B in melanocytes. The results are presented as the means ± standard deviations from triplicate experiments and statistically analysed by Mann-Whitney U test. *P < 0.05. Figure S2. The detection of GDF-15 in mucosal melanoma cells. (A) Western blotting was used to detect GDF-15. GAPDH is shown as a loading control. (B) The relative intensity of GDF-15 influenced by miR-let-7b, miR-let-7c and co-transfection with MDTH or CALU was quantified by software Image J software and analysed statistically. The relative intensity of GDF-15 was normalized to GAPDH. The results are presented as the means ± standard deviations from triplicate scans and statistically analysed by Mann-Whitney U test. *P < 0.05. Figure S3. Immunohistochemistry for CALU in mucosal melanoma tissues. CALU protein from mucosa, mucosal nevi and mucosal melanoma patients was stained by immunohistochemistry. CALU protein staining was strongly positive in mucosal melanoma, weakly positive in mucosal nevi and negative in mucosal tissue. (ZIP 2193 kb) [file 13046_2019_1190_MOESM1_ESM.zip › supplement-2.tif]

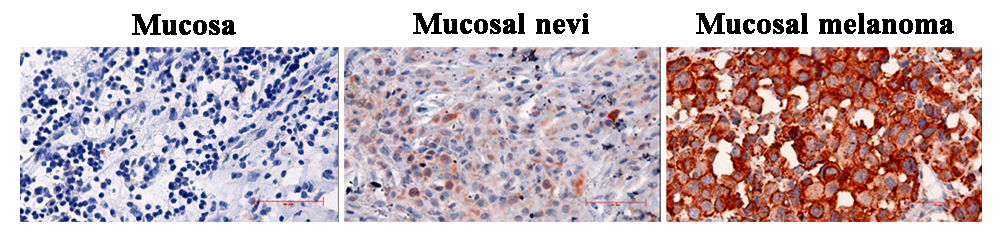

Supplement: Supplementary file 1 — Figure S1. The increased expression of miR-let-7b and miR-let-7c in mucosal melanoma cells. The expression of miR-let-7b (A) and miR-let-7c (B) was upregulated in HMVII cells through transient transfection. The expression of miR-let-7b (C) and miR-let-7c (D) was upregulated in GAK cells through transient transfection. The expression of miR-let-7b (E) and miR-let-7c (F) was upregulated in HMVII cells through virus infection. The expression was detected through qPCR with an internal control RNU6B in melanocytes. The results are presented as the means ± standard deviations from triplicate experiments and statistically analysed by Mann-Whitney U test. *P < 0.05. Figure S2. The detection of GDF-15 in mucosal melanoma cells. (A) Western blotting was used to detect GDF-15. GAPDH is shown as a loading control. (B) The relative intensity of GDF-15 influenced by miR-let-7b, miR-let-7c and co-transfection with MDTH or CALU was quantified by software Image J software and analysed statistically. The relative intensity of GDF-15 was normalized to GAPDH. The results are presented as the means ± standard deviations from triplicate scans and statistically analysed by Mann-Whitney U test. *P < 0.05. Figure S3. Immunohistochemistry for CALU in mucosal melanoma tissues. CALU protein from mucosa, mucosal nevi and mucosal melanoma patients was stained by immunohistochemistry. CALU protein staining was strongly positive in mucosal melanoma, weakly positive in mucosal nevi and negative in mucosal tissue. (ZIP 2193 kb) [file 13046_2019_1190_MOESM1_ESM.zip › supplement-3.tif]
