# Supplementary material for: miR-let-7b and miR-let-7c suppress tumourigenesis of human mucosal melanoma and enhance the sensitivity to chemotherapy
Source: J Exp Clin Cancer Res. 2019 May 22;38:212. doi: 10.1186/s13046-019-1190-3 (PMC6532197; doi:10.1186/s13046-019-1190-3)
Supplement: Supplementary file 2 — Table S1. Antibodies used in this study. Table S2. Origins and genetic aberrations of mucosal melanoma cells used in this study. Table S3. Clinical characteristics of established PDX models from mucosal melanoma patients used in this study. Table S4. Kaplan-Meier analysis of clinical characteristics of DFS. Table S5. Kaplan-Meier analysis of clinical characteristics of PFS in temozolomide-based chemotherapy. Table S6. Kaplan-Meier analysis of clinical characteristics of PFS in paclitaxel-based chemotherapy. Table S7. Multivariate cox regression analysis of potential predictors of PFS in paclitaxel-based chemotherapy. (ZIP 112 kb) [file 13046_2019_1190_MOESM2_ESM.zip › Supplementary Table S2-new.doc]

Table S2. Origins and genetic aberrations of mucosal melanoma cells used in this study

| Cells | Origin | BRAF | NRAS | CKIT | PDGFR |
| --- | --- | --- | --- | --- | --- |
| HMVII | Vaginal | G469V | Q61K | WT | WT |
| GAK | LN (Vaginal) | WT | Q61L | WT | WT |
| The mutations were detected by Sanger Sequencing after PCR. BRAF mutations in these cell lines were in exon 15, NRAS mutations in these cell lines were in exon 2. LN (Vaginal): Inguinal lymph node of a vaginal melanoma; WT: wild type. | | | | | |
