# Supplementary material for: miR-let-7b and miR-let-7c suppress tumourigenesis of human mucosal melanoma and enhance the sensitivity to chemotherapy
Source: J Exp Clin Cancer Res. 2019 May 22;38:212. doi: 10.1186/s13046-019-1190-3 (PMC6532197; doi:10.1186/s13046-019-1190-3)
Supplement: Supplementary file 2 — Table S1. Antibodies used in this study. Table S2. Origins and genetic aberrations of mucosal melanoma cells used in this study. Table S3. Clinical characteristics of established PDX models from mucosal melanoma patients used in this study. Table S4. Kaplan-Meier analysis of clinical characteristics of DFS. Table S5. Kaplan-Meier analysis of clinical characteristics of PFS in temozolomide-based chemotherapy. Table S6. Kaplan-Meier analysis of clinical characteristics of PFS in paclitaxel-based chemotherapy. Table S7. Multivariate cox regression analysis of potential predictors of PFS in paclitaxel-based chemotherapy. (ZIP 112 kb) [file 13046_2019_1190_MOESM2_ESM.zip › Supplementary Table S3.Clinical characteristics.doc]

Table S3. Clinical characteristics of established PDX models from mucosal melanoma patients used in this study

| Code | Gender | Age | Stage | Survival time (month) | Survival status | miR-let-7b | miR-let-7c | BRAF | NRAS | CKIT | PDGFR |
| --- | --- | --- | --- | --- | --- | --- | --- | --- | --- | --- | --- |
| PDX 1 | M | 51 | IV | 63.8 | 0 | High | High | WT | WT | WT | WT |
| PDX 2 | F | 63 | III | 7.6 | 1 | Low | Low | WT | WT | WT | WT |
| PDX 3 | M | 55 | IV | 7.9 | 0 | Low | High | WT | WT | WT | WT |
| PDX 4 | M | 65 | III | 29.0 | 1 | Low | Low | WT | WT | WT | WT |
| The expressions of miR-let-7b and miR-let-7c were quantified by qPCR and cutoff values of expression for miR-let-7b and miR-let-7c were 0.02452642 and 0.13255146. The mutations were detected by Sanger Sequencing after PCR and the detection for BRAF mutations were in exon 15; NRAS mutations were in exon 2, 3; CKIT mutations were in exon 9, 11, 13, 17, 18; PDGFR mutations were in exon 12, 14, 18. Gender: F, female; M, male; Survival status: “1” indicates survival; “0” indicates for death; WT, wild type. | | | | | | | | | | | |
